# Hjort-YOLO: a lightweight and explainable computer vision framework for wildlife detection

**DOI:** 10.3389/frai.2026.1930016

**Published:** 2026-10-14

**Authors:** Md Abdul Momin, Mehak Khan, Reza Arghandeh, Richard Bischof, Stein Joar Hegland, Terje Moen, Martin F. Stølen

**Affiliations:** 1 Department of Computer Science, Electrical Engineering and Mathematical Sciences, Western Norway University of Applied Sciences, Førde, Norway; 2 Department of Computer Science, Electrical Engineering and Mathematical Sciences, Western Norway University of Applied Sciences, Bergen, Norway; 3 Faculty of Environmental Sciences and Natural Resource Management, Norwegian University of Life Sciences, Ås, Norway; 4 Department of Civil Engineering and Environmental Sciences, Western Norway University of Applied Sciences, Sogndal, Norway; 5 SINTEF, Trondheim, Norway; 6 Western Norway Research Institute, Sogndal, Norway

**Keywords:** computer vision, deep learning, deer, wildlife, YOLO

## Abstract

**Introduction:**

Automated animal detection is an important component of ecological monitoring and may in the future be used also for wildlife vehicle collision mitigation by constructing efficient alert systems. However, the often complex environmental background in habitats along roads and different sizes due to the distance from the camera lens of animals in the images may reduce the detection accuracy, whereas high-precision detection requires computationally expensive object detection methods. This paper presents Hjort-YOLO, an efficient computer vision framework for detecting wildlife from camera-trap images acquired under challenging background conditions.

**Methodology:**

The proposed framework was developed by enhancing the lightweight YOLOv11n architecture through targeted modifications to the backbone and detection head by improving the cross-stage partial convolutional block (C2f-W), an area attention block (A2C2f-W), and reducing a detection head compared to the YOLOv11n algorithm, enabling an optimal balance between detection accuracy and computational efficiency. Hjort-YOLO is evaluated on the custom Svanøy deer dataset and the African Wildlife dataset, which include complex forest scenes, occlusions, background clutter, and motion blur.

**Results:**

Experimental findings indicate that the Hjort-YOLO achieves detection accuracy comparable to state-of-the-art object detection models while significantly improving runtime performance. Hjort-YOLO provides 0.2 percent, 13 percent, and 17 percent better mean average precision, frames per second, and inference time, respectively, while detecting wildlife compared to YOLOv11n. The computation complexity, such as Giga Floating Operations per Second, was reduced by 19 percent compared to the YOLOv11n algorithm.

**Discussion:**

These performance gains show the suitability of the proposed framework for detecting wildlife from ground-based sensors like camera traps, providing real-time performance and high detection accuracy in resource-constrained platforms.

## 1 Introduction

Wildlife monitoring has become an essential aspect of preserving ecosystems due to the decline in global biodiversity (Zhu et al., 2025). Biodiversity is declining globally because of land-use changes, climate change, pollution, and other threats (Díaz et al., 2019). Some reports point to more than 70 percent decline in wild mammal populations between 1970 and 2020 (WWF, 2024), which now make up 6 percent of all mammal biomass, whereas humans (36 percent) and livestock (58 percent) represent the rest (Greenspoon et al., 2023). Vital factors for the imbalance in biodiversity include climate change, deforestation, and the expansion of road networks, which lead to the decline of wildlife (Tøn et al., 2024). At the same time, wildlife vehicle collisions are a global issue of growing importance in both developing and developed countries. Animal-vehicle collisions (AVCs) also concern ecology and society since they are affected by the traffic volume, and affect the habitat of wildlife, with economic, ecological, and medical costs. There were 725,000 to 1,500,000 animal-vehicle collision cases reported to the police in the United States per year, and 4–8 animals are killed per day in Canada according to Nandutu et al. (2022). Collisions between vehicles and wildlife have long been on the rise, also in Europe, due to the increase in ungulate populations, as well as the high traffic density on the roads in Europe (Rolandsen et al., 2011). In 2025, more than 14,500 moose and deer were killed on Norwegian roads and railways (Landbruksdirektoratet, 2026).

Hence, reducing animal-vehicle collisions has become a global issue to minimize casualties and preserve the ecological balance. Automated wildlife monitoring provides critical information about animal populations, distribution, and behavior that is essential for protecting the ecological balance. The traditional wildlife monitoring requires manual labor techniques for direct observation, transect surveys, and marker-based capturing methods. As camera traps, drones, and remote sensing become more widespread, the volume of visual data collected far exceeds what can be annotated manually in a timely manner. However, there are challenges related to data accuracy and reliability when following these methods, which can lead to bias during observation and disturb the animal behavior (Yang et al., 2024). Deep learning-based object detection offers a promising solution to this problem: by training models to automatically detect animals in images or video frames, researchers can accelerate data processing and scale biodiversity monitoring. This can also support the development of automatic systems for detecting animals near roads, from ground-based sensors to drones, which can potentially be used to warn drivers and help reduce animal-vehicle collisions. The work presented here is part of the WILDETECT project, which aims to explore technologies related to reducing animal-vehicle collisions in Norway.

Recent research works (Huang et al., 2024; Lu and Lu, 2023; Roy et al., 2023) have shown that even relatively lightweight YOLO-based models can effectively identify and classify animals from camera-trap images, significantly reducing manual effort. Animal monitoring applications using artificial intelligence have been proposed for livestock farming as well as wildlife recognition (Cabezas et al., 2022). However, monitoring wild animals requires substantial and continuous data acquisition techniques for processing and recognizing the animals. Machine learning algorithms can learn from large amounts of data to recognize the patterns of animals (Tuia et al., 2022). Computer systems, along with object detection techniques, can provide information about the location of the animals as well as their species. The performance of object detection methods has reached a high level using convolutional networks. The classic object detection algorithms used moving window mechanisms to get the region proposals, which were sent to the classifiers for extracting feature maps and classifications. However, the classic object detection algorithms did not provide high accuracy on diverse object-based datasets such as ImageNet (Russakovsky et al., 2015) and COCO (Lin et al., 2014).

Deep Neural Network (DNN) models were developed by researchers to extract feature maps more accurately (Ibraheam et al., 2023). Pascal Visual Object Classes (VOC) (Everingham et al., 2015) was the first DNN algorithm based on region proposals, and Girshick et al. (2014) merged region proposals as a Convolutional Neural Network (CNN). The existing object detection approaches are generally classified into two categories, single-stage and two-stage object detection. In two-stage object detection algorithms, the generation of regions of interest (RoIs) is performed in the first stage, with regional classification and location refinement to predict the target object performed in the second stage (Du et al., 2020). The most common two-stage object detection methods are R-CNN (Region-based Convolutional Neural Network) (Girshick et al., 2014), Fast R-CNN (Girshick, 2015), Faster R-CNN (Ren et al., 2017), and Mask R-CNN (He et al., 2017).

A CNN-based two-stage object detection algorithm was developed by Hans et al. (2020) to detect deer using a dash-cam in the car for traffic safety, where the target objects, including deer, were localized using the sliding window method. In a similar study, a hybrid approach combining the Histogram of Oriented Gradients (HOG) and CNN model was used for detecting nocturnal animals on the roadsides for road traffic safety applications (Munian et al., 2022). R-CNN, Libra R-CNN, and RetinaNet (Lin et al., 2017) were used in a diverse animal dataset study where the performance of these three object detection algorithms was compared to detect animals in the open savanna or sparsely wooded areas for wildlife detection (Delplanque et al., 2022). However, since the detection process is performed in two stages, it is computationally complex and requires more time for training.

A single Convolutional Neural Network (CNN) can also be used in a one-stage object detection method to predict the position and classification of the target objects in one go. The one-stage object detection methods are more time-efficient than two-stage methods because classification and regression are performed in a single stage. One-stage object detection methods are preferred for real-time object detection applications. The most common one-stage object detection algorithms are Single Shot Detector (SSD) (Liu et al., 2016), RetinaNet (Lin et al., 2017), RefineDet (Zhang et al., 2018), and You Only Look Once (YOLOv1) (Redmon et al., 2016), YOLOv11 (Jocher and Qiu, 2024), and YOLOv12 (Tian et al., 2025).

Object detection algorithms are being specialized for various applications, among other animal detection and monitoring. A channel enhancement (CE) method was applied in RetinaNet (CE-RetinaNet), where the CE module extracted the feature maps of the infrared images more accurately to detect elephants (Zhang and Cai, 2023). CE-RetinaNet algorithm filtered out the occlusion, and the localization capability was improved as well. RetinaNet was also used by Delplanque et al. (2022) on multiple large animals where RetinaNet provided less detection accuracy than other two-stage object detection algorithms. The series of YOLO algorithms is popular among researchers for real-time target object detection. YOLO algorithms are used in animal detection as well. The YOLOv2 algorithm was improved for the wildlife-vehicle collision mitigation system by Ibraheam et al. (2023).

YOLOv5 was used for wildlife monitoring and comparing the false positive and false negative samples with manual detection in Povlsen et al. (2023). However, YOLOv5 missed several false positive and false negative samples while detecting the target animals in this study. In a similar study (Xiong et al., 2024), the authors improved YOLOv5 by proposing the AD-YOLOv5 algorithm, which simplified the feature map extraction method and improved bounding box orientation for predicting animals. However, AD-YOLOv5 was designed for detecting only the key body parts of sika deer in the images. There are numerous machine learning and deep learning-based algorithms available for detecting a wide range of objects from the ground-level perspective. However, there is no specific object detection framework for detecting camera-trap wildlife while maintaining the minimal trade-off between detection accuracy and computation complexity for real-time detection. In this study, we have improved the YOLOv11n algorithm to detect wildlife captured from camera traps. The algorithm maintains the detection accuracy and inference time for detecting wildlife. The contribution of this study is summarized as follows:

- This study aims to develop a wildlife detection framework, Hjort-YOLO, based on YOLOv11n using camera trap images from Svanøy Island, Norway.
- The computational complexity of the proposed algorithm was reduced by extricating a detection head. A2C2f (Area-Attention Cross-Stage Partial fusion) and C2f blocks were modified for balancing the trade-off between detection accuracy and detection time.
- Explainable Artificial Intelligence (XAI) tool Captum was integrated with the proposed framework to improve the interpretability of wildlife detection, enabling visualization of discriminative spatial regions in the image to provide insight into the framework's decision-making process.

## 2 Methodology

In this section, the Wildlife image collection is described in Section 2.1, the wildlife detection framework overview in subsection 2.2, and the proposed Hjort-YOLO model architecture in Section 2.3. Sections 2.4 and 2.5 describe the Wildlife Segmentation with SAM2 and Wildlife visualization with XAI, respectively. Hence, these Sections are a continuation of the same study. Finally, Section 2.6 represents the training environment maintained in this study for all the experimental settings.

### 2.1 Wildlife image collection

In this research, one custom dataset (Svanøy deer dataset) and one public dataset, African Wildlife (Ferreira, 2024), were used. Both of the image datasets were captured using ground cameras where the target animals were captured at relatively close range and occupied a large portion of the image frame in most of the images.

#### 2.1.1 Use case A: Svanøy deer dataset

The Svanøy deer dataset consists of a single class of deer, which is red deer (*Cervus elaphus*), and the images were captured using 60 cameras on Svanøy Island (61.49061°, 5.1451796°) in Norway. The images used in this study were obtained from Browning trail cameras (DarkOps HD Pro BTC-6HDPX, BTC-6HDP, BTC-PXD, and Spec Ops Full HD) equipped with infrared flash for nighttime imaging. These cameras do not record thermal images, and therefore, no thermal color scheme (e.g., white-hot or black-hot) was used. The dataset included both daytime RGB images and nighttime near-infrared (NIR) monochrome images acquired under infrared illumination. Data augmentation was not performed to create the Svanøy deer dataset. There are 3,183 images, with 1,432 RGB images and 1,751 near infrared (NIR) images. The images were labeled manually using Roboflow (Dwyer et al., 2026), and the dataset was split into three parts, with ratios of 80 percent, 10 percent, and 10 percent for training, validation, and testing, respectively. Figure 1a shows some samples of annotated deer photos in the Svanøy deer dataset.

**Figure 1:**
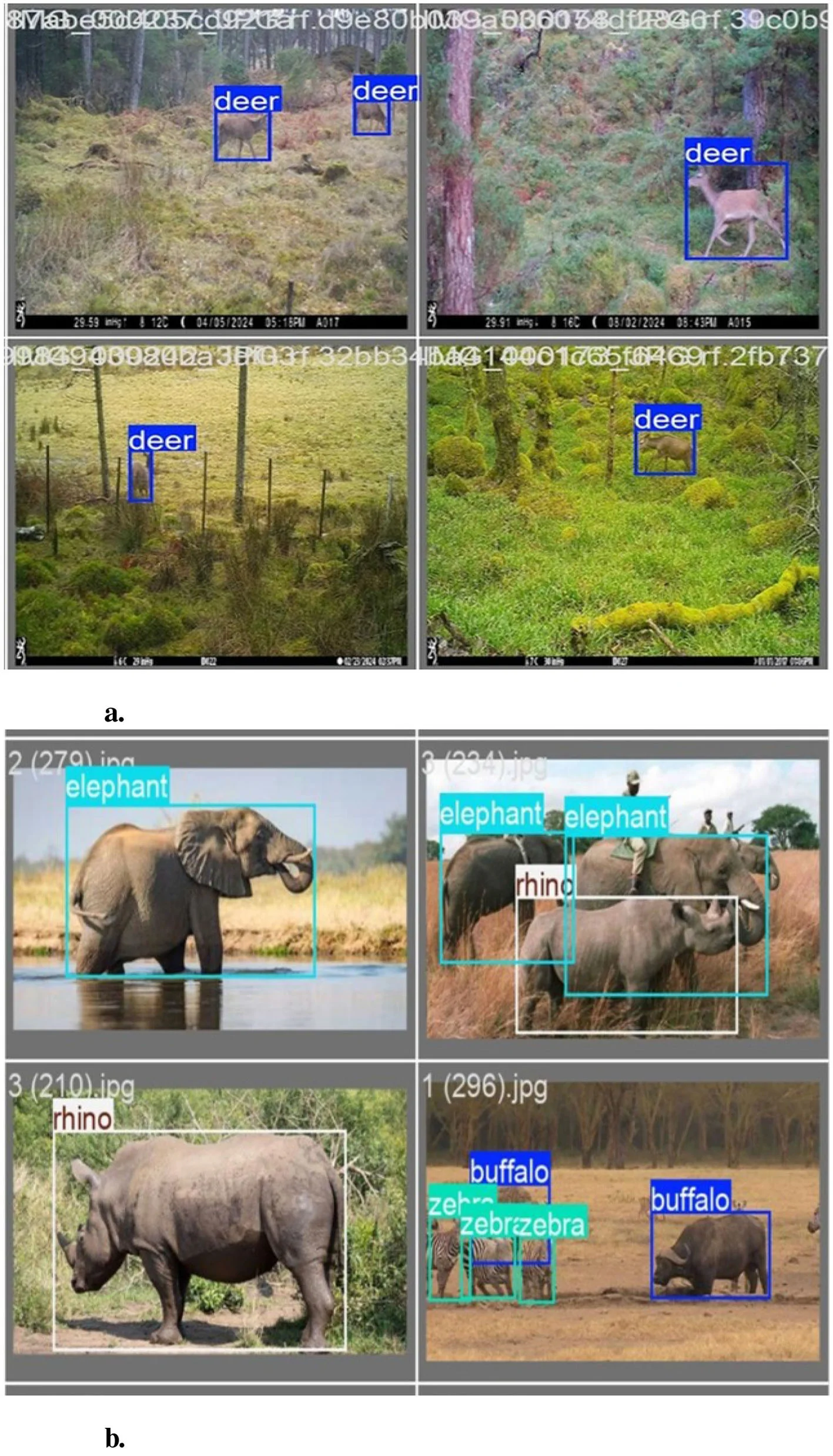
Annotation on images in the **(a)** Svanøy deer dataset (adapted with permission from the ‘deer open dataset Computer Vision Dataset', https://universe.roboflow.com/somethings/deer-open-dataset-8cx4z, licensed under CC-BY 4.0). **(b)** African wildlife dataset (adapted with permission from Ferreira, B. (2024), https://docs.ultralytics.com/, licensed under the GNU Affero General Public License v3.0).

#### 2.1.2 Use case B: African Wildlife dataset

In this study, a public dataset named African Wildlife was used, where there are 1,504 RGB images with four different animals named buffalo, elephant, rhino, and zebra (Figure 1b). The annotated file was downloaded from Ferreira (2024), and the annotation was manually adjusted using Roboflow. The dataset was split into train, validation, and test images by 80 percent, 10 percent, and 10 percent, respectively. In this task, all the African wildlife images were RGB images (Table 1).

**Table 1:** African wildlife dataset.

Classes	Train image	Valid image	Test image	Total
Buffalo	265	62	49	376
Elephant	268	51	57	376
Rhino	265	56	55	376
Zebra	254	56	66	376
Total	1,052	225	227	1,504

### 2.2 Wildlife detection framework overview

This study presents a wildlife detection and visualization framework using ground camera-captured images (Figure 2). The core of our wildlife detection model is based on the YOLOv11n algorithm for detecting wildlife, by maintaining good detection accuracy and low inference time. The framework was divided into three blocks. Initially, the dataset was prepared from the camera traps, which consisted of RGB and night vision images. Secondly, the YOLOv11n architecture was modified by improving the backbone and reducing the number of detection heads. Subsequently, the dataset was passed through the improved YOLOv11n algorithm, and it was trained from scratch without pretrained weights to detect wildlife. Additionally, this study emphasizes the importance of visualizing detected animals through advanced segmentation techniques and Explainable Artificial Intelligence (XAI). This visualization process enhances the interpretability of the model's decision-making in detection and classification, while helping to understand the mechanism of wildlife detection. Image segmentation and interpretability using XAI were performed as post-processing steps after successful detection of the target animals in the images, offering a more comprehensive understanding of the model's performance. The proposed Hjort-YOLO consists of a single neural network, which was used for training both Use case A and Use case B in this study.

**Figure 2:**
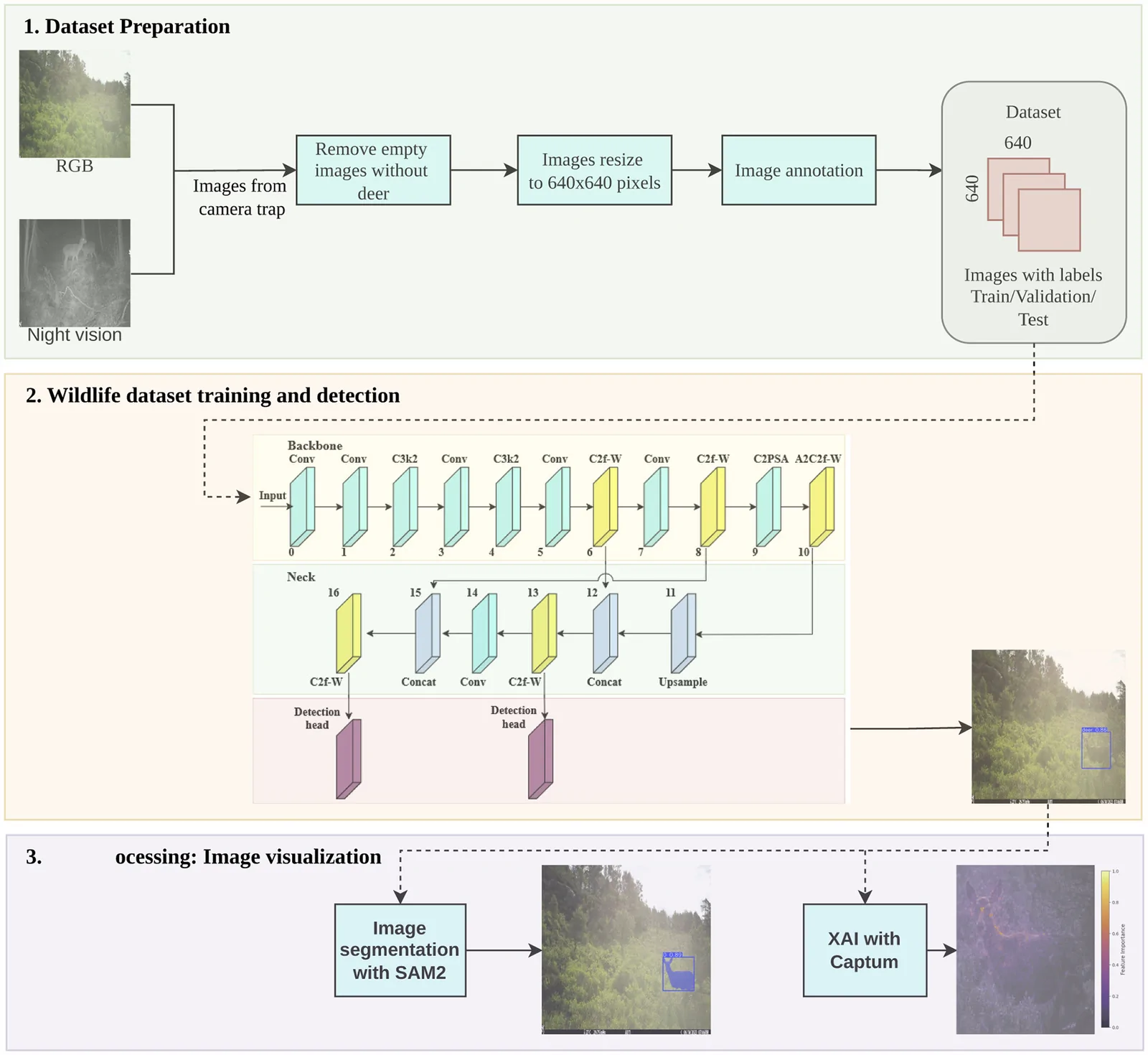
Wildlife detection and visualization framework overview (adapted with permission from the ‘deer open dataset Computer Vision Dataset', https://universe.roboflow.com/somethings/deer-open-dataset-8cx4z, licensed under CC-BY 4.0).

### 2.3 Proposed Hjort-YOLO model architecture

The proposed Hjort-YOLO algorithm (Figure 3) was developed based on the YOLOv11n model. YOLOv11n (YOLOv11-nano) is the fastest and lightweight version of YOLOv11 for detection purposes compared to the small, medium, large, and extra-large versions. YOLOv11n is suitable for real-time applications, which provides competitive detection accuracy, inference speed, and computational efficiency (Huang et al., 2024). YOLOv11 shows versatile performance in object detection, classification, pose estimation, and segmentation tasks. The three major components of the YOLOv11 architecture are the backbone, neck, and prediction head. Feature maps from the input image are extracted by the backbone through various convolutional layers. The residual connections and attention mechanism in the backbone improve feature extraction by allowing the spatial dimensions to be reduced and the depth of the feature maps to be increased.

**Figure 3:**
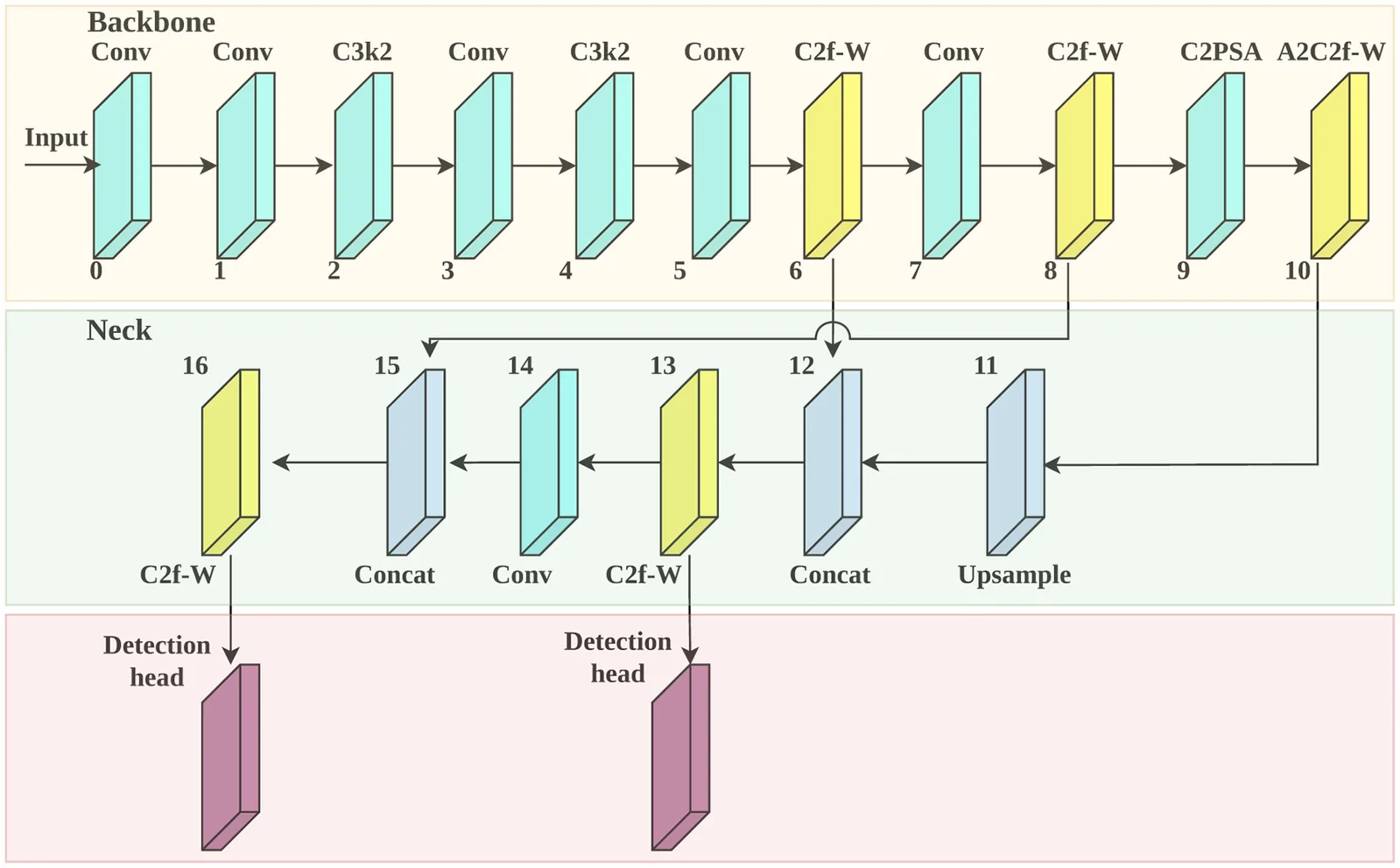
Proposed Hjort-YOLO model architecture.

The neck creates a link between the backbone and the head while enhancing the feature maps for improved object detection. Feature Pyramid Networks (PANs) improve feature map extraction by fusing information across multiple scales for object detection of varying sizes. The head is responsible for predicting the target object with object localization and bounding box creation using the regression method (Khanam and Hussain, 2024). The major improvements in the YOLOv11 architecture are the inclusion of the C2PSA (Convolutional block with Stage Partial Attention), SPPF (Spatial Pyramid Pooling-Fast), and C3k2 (Cross-Stage Partial Block with kernel size 2) modules (Khanam and Hussain, 2024). These blocks reduce the algorithm's computational complexity, enabling the model to be trained more quickly. The main differences among different versions of the YOLOv11 algorithm are the number of repeated blocks (network depth), channels per layer (channel width), and maximum allowable channels. The reduced depth and width multiplier in terms of the blocks and layers in the architecture makes YOLOv11n suitable for detecting target objects in real-time in computationally constrained edge devices.

The proposed Hjort-YOLO algorithm was designed to reduce the computational complexity by maintaining the detection accuracy and precision. Besides, the objective was to efficiently integrate multi-scale spatial features from the images to improve wildlife detection in complex natural backgrounds. The images go through the backbone as input, and the backbone begins with a sequence of convolutional layers that gradually expand the receptive field. The alternating convolutional blocks (Conv and C3k2) enhance the early and mid-level feature extraction in the backbone. The C3k2 block was introduced in the YOLOv11 algorithm to make the algorithm computationally efficient and faster in processing the feature extraction process in the architecture (Khanam and Hussain, 2024). C3k2 block contains two smaller convolutions in place of one large convolutional block, and it operates with a smaller kernel size to reduce the processing speed. The C2f-W block inspired from C2f block in YOLOv8 (Varghese and Sambath, 2024) refined the channel interaction and gradient propagation for higher-level representation (Figure 4). The input goes through a 1 × 1 convolution layer, and the feature maps are split into two streams. The second feature stream goes through two bottleneck blocks, which produce intermediate features. The concatenation layer added the direct feature streams and the output features of the two bottleneck blocks. The equation of dense gradient paths in the C2f-W block is represented by:


$$\begin{array}{c} \frac{\partial O}{\partial U_{2}}=\overset{n}{\underset{k=0}{\sum }}\frac{\partial O}{\partial Y_{k}}\frac{\partial Y_{k}}{\partial U_{2}} \end{array}$$ (1)

In this Equation 1, *k* represents the bottleneck index, and *n* represents the number of bottlenecks in the C2f-W architecture. The second split feature stream, which is processed by bottleneck layers, is denoted by *U*_2_, the output of the *k*-th bottleneck and first feature split transformation is represented by *Y*_*k*_, and the final fused output of the C2f-W is represented by *O*. In the Hjort-YOLO architecture, the C3k2 block was replaced by the C2f-W block in the lower backbone and the neck area, which increased the number of trainable parameters. In Hjort-YOLO, the C2f-W block has more trainable parameters than the C3k2 block, because the C2f-W block relies heavily on channel expansion and large 1 × 1 fusion convolutions that dominate parameter count. This large tensor is then projected back to the output dimension via another expensive 1 × 1 convolution, leading to parameter growth that scales with both the expansion ratio and the number of bottlenecks.

**Figure 4:**
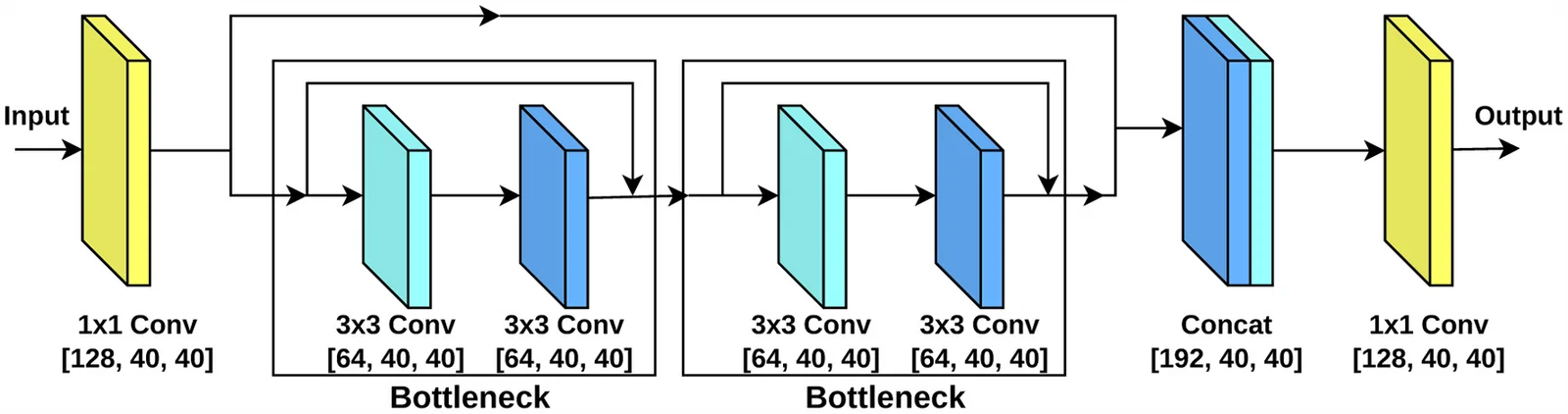
C2f-W architecture.

The C2f-W block maintains the same mathematical and computational formulation compared to the C2f block in YOLOv8. However, the C2f-W block normalizes and validates all hyperparameters in the architecture, such as group sizes, expansion ratios, and the depth of the bottleneck. The normalization of these parameters prevents the tensor shape inconsistencies while extracting and formulating the feature maps of the image. It also prevents the runtime failure and increases the reliability and structural safety in the Hjort-YOLO architecture during model initialization.

In the Hjort-YOLO network, a C2PSA block at the terminal stage introduces a lightweight partial self-attention mechanism to strengthen global contextual modeling with minimal computational overhead. This attention module enhances the architecture by identifying complex and partially occluded target objects in the images, which is quite common in the object detection task (Khanam and Hussain, 2024). The C2PSA block refines the spatial extractions, improving the network's performance in images with complex visual environments. YOLOv11 has a Spatial Pyramid Pooling-Fast (SPPF) block at the end of the backbone, which is a lightweight feature-aggregation module. The SPPF module expands the architecture's receptive field without significantly increasing the computational cost. The SPPF module was replaced by a A2C2f-W block in the Hjort-YOLO architecture to improve the feature extraction and detection performance, which increased the computational complexity of the model.

Figure 5 shows the A2C2f-W architecture, which was inspired by the YOLOv12 algorithm (Tian et al., 2025). The YOLOv12 architecture integrates attention mechanisms, and it replaces the convolutional transformations, enabling more effective spatial modeling while maintaining computational efficiency. The Area-Attention Block (ABlock) is designed to enhance spatial reasoning by computing attention not at the individual pixel level, but over predefined areas of the feature map. The input tensor is partitioned into non-overlapping regions instead of treating each spatial location independently, enabling the model to capture mid-range contextual relationships while minimizing the high computational cost of full self-attention. A2C2f-W relies on lightweight multi-branch feature extractors, where each C2f-W block expands the input tensor with 1 × 1 convolution, applies a sequence of transformations on the reduced feature space, concatenates the intermediate outputs, and fuses them back into the original channel dimension. In the A2C2f-W block, the attention for each spatial area index *m* is computed as (Chandrashekhar et al., 2025):


$$\begin{array}{c} Q_{m}=W_{Q}U_{1}^{\left(m\right)} \end{array}$$ (2)

$$\begin{array}{c} K_{m}=W_{K}U_{1}^{\left(m\right)} \end{array}$$ (3)

$$\begin{array}{c} V_{m}=W_{V}U_{1}^{\left(m\right)} \end{array}$$ (4)

$$\begin{array}{c} A_{m}=\text{Softmax}\left(\frac{Q_{m}K_{m}^{T}}{\sqrt{d}}\right)V_{m} \end{array}$$ (5)

In Equations 2–5, $U_{1}^{\left(m\right)}$ is represented by the feature map corresponding to the *m*-th spatial area. *W*_*Q*_, *W*_*K*_, and *W*_*V*_ are denoted by learnable query, key, and value projection matrices, respectively. *Q*_*m*_, *K*_*m*_, and *V*_*m*_ are represented by query, key, and value matrix for area m in the area attention module. The resulting attention output for the *m*-th area is denoted by *A*_*m*_, with embedding dimension *d*. The A2C2f-W module enhances the stability and compatibility of the area-attention C2f-W block. The proposed modification preserves the original architecture's efficiency while improving gradient stability, model convergence, and integration within the Hjort-YOLO framework. The proposed A2C2f-W module builds upon the original Area-Attention C2f design by refining its residual formulation and implementation structure.

**Figure 5:**
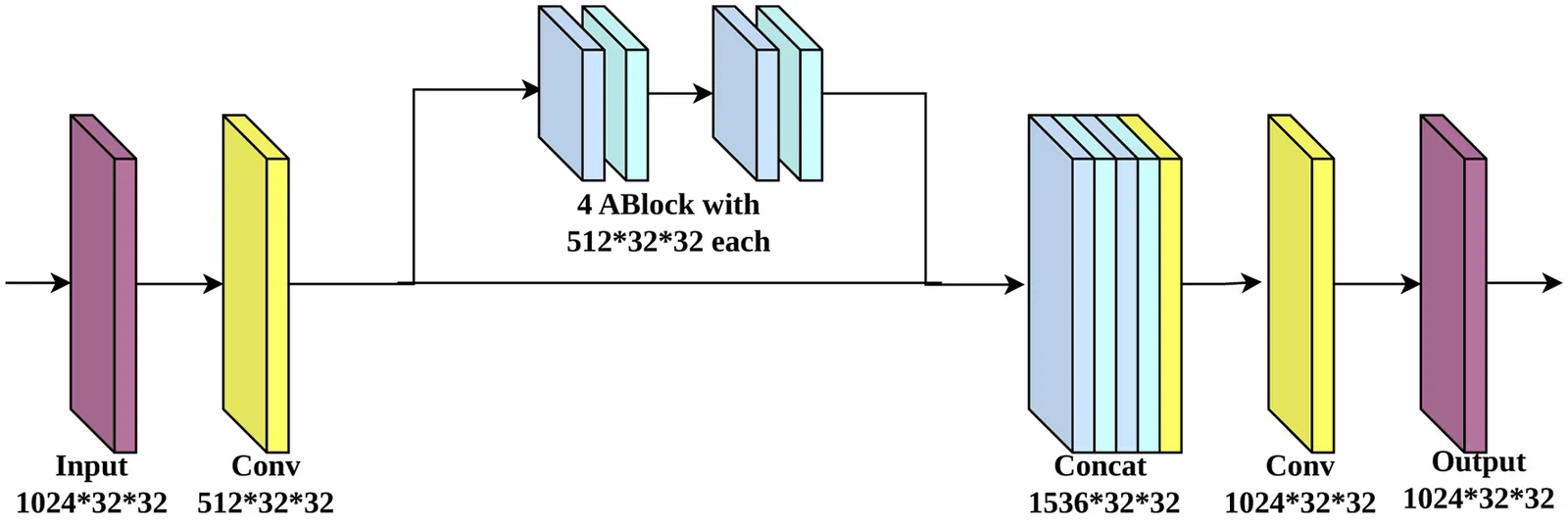
A2C2f-W architecture.

One of the major differences between YOLOv11n and Hjort-YOLO is the number of detection heads. YOLOv11 has three detection heads, whereas Hjort-YOLO has two detection heads. For a 640 × 640 image input size, the output image sizes in YOLOv11n detection heads are 20 × 20, 40 × 40, and 80 × 80 pixels. The detection heads with 20 × 20, 40 × 40, and 80 × 80-pixel output image sizes are responsible for detecting large, medium, and small target objects, respectively, in the images. In this study, the wildlife were captured using ground cameras. Hence, the target objects are comparatively large in the images. This study focuses on detecting large target animals in the frame as the cameras can be mounted near the road for reducing the animal-vehicle collisions. The detection head 80 × 80 was removed from the architecture to reduce the computational parameter significantly. The addition of the C2f-W and A2C2f-W block in the architecture increased the computational parameters and detection accuracy with enhanced feature maps extractions. In contrast, the reduction of one detection head balanced the minimal trade-off between detection accuracy and computational complexity of the architecture.

### 2.4 Wildlife segmentation with SAM2

In this study, the visualization after wildlife detection was evaluated using the Segment Anything Model 2 (SAM2) (Ravi et al., 2024). SAM2 is the improved version of the SAM model (Kirillov et al., 2023). SAM uses zero-shot segmentation without the need for training to segment, whereas the SAM2 model improves the accuracy, functionality, and speed for segmenting in complex backgrounds and challenging images. One of the major differences between SAM and SAM2 is the ability to segment videos by SAM2. In SAM2, multiple memory attention layers, a memory encoder, and a memory bank are used for improving the performance (Kirillov et al., 2023). Here SAM2 is used for segmenting the wildlife for both the Svanøy deer and the African Wildlife dataset. The images were processed through YOLOv11n and Hjort-YOLO algorithms, and the predictions were made with a bounding box and confidence score (class probability) using neural regression. The bounding boxes acted as geometric prompts for SAM2, and it performed a conditional mask on the target object at the bounding box. In SAM2, the image goes through a vision transformer, and the bounding box goes through a learned projection, and these are fused through a cross-attention decoder for producing a pixel-level mask for the segmentation. Hjort-YOLO and SAM2 work as a two-stage detection-segmentation process, where the Hjort-YOLO algorithm provides fast and efficient target object localization, and SAM2 refines the detection into pixel-accurate instance segmentation.

### 2.5 Wildlife visualization with XAI

In this study, the wildlife detection was visualized using Gradient-weighted class activation mapping (Grad-CAM) (Selvaraju et al., 2017) and Captum (Kokhlikyan et al., 2020). These XAI tools interpret the focus area through a heatmap in the image while detecting the target object as post-processing of the wildlife images. Grad-CAM was integrated with Hjort-YOLO to provide visual interpretability by emphasizing the regions of the input image that most influence the model's predictions. Grad-CAM focuses on the final or assigned convolutional layer to create a localization map of the target class in the model. The localization map of Grad-CAM on class *c* is calculated as (Selvaraju et al., 2017):


$$\begin{array}{c} L_{\text{Grad}\text{-CAM}}^{c}=\text{ReLU}\left(\underset{k}{\sum }\alpha_{k}^{c}A^{k}\right) \end{array}$$ (6)

$$\begin{array}{c} \alpha_{k}^{c}=\frac{1}{Z}\underset{i,j}{\sum }\frac{\partial y^{c}}{\partial A_{i,j}^{k}} \end{array}$$ (7)

In Equations 6, 7, $L_{\text{Grad}\text{-}\text{CAM}}^{c}$ is for the localization map, $\alpha_{k}^{c}$ denotes weights that are important features of the map *A*^*k*^ for class *c*. Activation at spatial location (*i, j*) in the *k*-th feature map is denoted by $A_{i,j}^{k}$. The feature map weight is calculated using the normalization of spatial dimensions and the output score *y*^*c*^ from class *c*. The Rectified Linear Unit (ReLU) activation function only focuses on the positive target score in the map for localization. Hjort-YOLO extracts feature maps of the images by using a deep convolutional backbone, and the detection head provides output with bounding boxes, objectness score, and class probabilities. Grad-CAM examines the gradients of a specific prediction and flows back through the final convolutional layers of the backbone. The gradients specify the spatial features to decide the prediction of the Hjort-YOLO model. In this study, Grad-CAM used the last convolutional layer of the Hjort-YOLO model to visualize feature maps of wildlife.

Besides Grad-CAM, the Captum tool interprets machine learning models in a user-friendly way while dealing with images and texts (Khan et al., 2024). One of the major advantages of the Captum tool is that it is compatible with numerous PyTorch models, and it requires minimal changes in the original neural network. The Hjort-YOLO model was integrated with the Captum tool naturally as a standard PyTorch model. Captum incorporates attribution and interpretability methods such as Integrated Gradients, GradientShap, DeepLift, and Occlusion, which provide a clearer visual understanding of the regions of the input image that influence the Hjort-YOLO algorithm's prediction. The Integrated Gradients tool function is defined as (Kokhlikyan et al., 2020):


$$\begin{array}{l} \text{IntegratedGradient}{\text{s}}_{i}\left(x\right)=\\ \;\left(x_{i}-x_{i}^{\prime }\right)\int_{0}^{1}\frac{\partial F\left(x^{\prime }+\alpha \left(x-x^{\prime }\right)\right)}{\partial x_{i}}d\alpha \end{array}$$ (8)

In Equations 8, IntegratedGradients_*i*_(*x*) represents the *i*-th feature of the input *x* attribution, the value and the baseline value of the *i*-th feature are represented by *x*_*i*_ and $x_{i}^{\prime }$ respectively, the model function is defined by *F*, the input from the baseline *x*′ to the output *x* is scaled by a scalar α. The gradient of the model output with respect to the *i*-th input feature is denoted as $\frac{\partial F\left(x^{\prime }+\alpha \left(x-x^{\prime }\right)\right)}{\partial x_{i}}$. The Captum tool can be integrated with any part of the Hjort-YOLO algorithm for computing the attribution score by focusing on the backbone feature maps or the final detection layer output. The visualization provides the important image features that contribute to bounding box localization or objectness confidence score. In this study, the predicted bounding boxes from Hjort-YOLO were subjected to output attribution analysis by the Captum tool. Captum backpropagates through the Hjort-YOLO algorithm to gather information about which regions or pixels in the bounding box influenced the prediction by using the corresponding objectness score. The output attribution maps can be visualized by overlaying a heatmap on the bounding box, showing the meaningful object structures, such as wildlife. It can also help identify irrelevant background information, which can significantly affect false-positive decisions when predicting bounding boxes. The integration between Hjort-YOLO and Captum can provide deeper information about the object detection decision-making process, which can help improve the model's architecture and support in bias detection.

### 2.6 Training environment and evaluation metrics

The experiments were carried out on a Google Colab (Google LLC, Mountain View, CA, USA) platform using a Tesla T4 GPU with 15 GB of memory, 2 virtual CPUs, and 12.7 GB RAM. The software environment was configured with Python 3.12.12, PyTorch 2.9.0 (Meta Platforms, Inc., Menlo Park, CA, USA) (Paszke et al., 2019) with CUDA 12.6 (NVIDIA Corporation, 2024), and Ultralytics version 8.3.232 (Jocher et al., 2023). To enable model interpretability, the experiments utilized the Captum and GradCAM libraries. The required packages, including numpy (v1.26.4) and torchcam (v0.4.1), were installed to support feature attribution and visualization of model predictions. In Ultralytics, the momentum was 0.95, weight decay 0.0005, batch size 16, initial learning rate 0.01, epochs 300, patience 20 (early stopping), image size 640 × 640, and the optimizer was SGD (Stochastic Gradient Descent).

In this study, the object detection parameters have been assessed by standard evaluation metrics (Ferri et al., 2009), which are precision (P), recall (R), F-1 score, and mean average precision (mAP). True positive (TP), false positive (FP), false negative (FN), and true negative (TN) were used to obtain the confusion matrix (Roy et al., 2023). In the detection task, mAP50 is an important evaluation metric where the mean of AP is calculated at the intersection over union (IoU) greater than 0.5. The mAP50–95 is evaluated with the average accuracy of IoU values between 0.5 and 0.95, which is a stricter threshold than mAP50. The equations of precision, recall, F1-score, and mAP are calculated as below (Equations 9–12) (Bhagabati et al., 2023):


$$\begin{array}{c} \text{Precision}\left(P\right)=\frac{TP}{TP+FP} \end{array}$$ (9)

$$\begin{array}{c} \text{Recall}\left(R\right)=\frac{TP}{TP+FN} \end{array}$$ (10)

$$\begin{array}{c} \text{F}1\text{-}\text{score}=\frac{2PR}{P+R} \end{array}$$ (11)

$$\begin{array}{c} \text{mAP}=\frac{1}{N}\overset{N-1}{\underset{i=0}{\sum }}\int_{0}^{1}P\left(R\right)dR \end{array}$$ (12)

## 3 Results and discussion

### 3.1 Use Case A: Svanøy dataset deer detection

In this study, the Svanøy deer dataset was used for training using multiple one-stage and two-stage object detection algorithms to compare the wildlife detection parameters (Table 2). RF-DETR (Roboflow Detection Transformer) Nano (Robinson et al., 2025) algorithm was trained on the Roboflow platform, and the other object detection algorithms were trained and evaluated on the Google Colab platform. RTDETR-l (Real Time Detection Transformer—large), YOLOv11m, YOLOv11n, and Hjort-YOLO algorithms were trained in the Ultralytics environment, with a patience of 20 for early stopping. Early stopping was set to converge the training process rapidly without overfitting, which helps improve generalization and computational efficiency. In this experiment, Faster R-CNN was chosen as a two-stage object detection algorithm, and RF-DETR Nano and RT-DETR were selected as transformer-based object detection algorithms. We selected YOLOv11n as the baseline lightweight model because its low computational complexity makes it a relevant comparison point for the Hjort-YOLO algorithm. YOLOv11-medium (YOLOv11m) was included as a higher-capacity model from the YOLOv11 series to compare the trade-off between computational complexity and detection performance. All models were trained using the same dataset split and image resolution as input to compare the evaluation metrics in this study.

**Table 2:** Object detection parameters on the Svanøy validation dataset.

Model	Epochs	Training time (h)	mAP	mAP (50–95)	Precision	Recall	F1-score	GFLOPS
Faster R-CNN (Ren et al., 2017)	50	7.02	0.938	–	0.6187	0.68	0.648	180
RF-DETR Nano (Robinson et al., 2025)	20	6.92	0.981	0.80	0.985	0.976	0.981	31.5
RT-DETR-l (Carion et al., 2020)	241	11.68	0.978	0.782	0.949	0.95	0.95	108
YOLOv11m (Khanam and Hussain, 2024)	98	3.253	0.982	0.803	0.966	0.936	0.951	68.2
YOLOv11n (Khanam and Hussain, 2024)	289	5.043	0.979	0.796	0.969	0.945	0.957	6.4
Hjort-YOLO	299	4.949	0.981	0.783	0.975	0.95	0.962	5.1

Mean average precision (mAP) is one of the major evaluation metrics in object detection as it measures how well the objects are detected and accurately localized in the image (Henderson and Ferrari, 2016). Hence, the mAP was the initial focus for benchmarking the object detection algorithms, and can be seen in Table 2. The two-stage object detection algorithm, Faster RCNN, achieved a moderate mAP of 0.938. The best mAP among the benchmarked object detection algorithms was displayed by YOLOv11m, at 0.982. Indeed, it was observed that all the object detection algorithms provided mAP around 0.98, except Faster RCNN. The Hjort-YOLO algorithm was trained ten times using the same dataset split, training configuration, and evaluation procedure on Svanøy deer dataset to evaluate the robustness against stochastic variation. The mean mAP of Hjort-YOLO was 0.9804 ± 0.004 and the mean mAP(50–95) was 0.7809 ± 0.0073. The mean precision, recall, and F1-score were 0.9775 ± 0.0085, 0.9518 ± 0.0076, and 0.9645 ± 0.0064, respectively. The standard deviation of each object detection parameter was relatively small. This indicates that Hjort-YOLO provided consistent detection performance despite stochastic variations across multiple training.

RF-DETR Nano is a transformer-based object detection algorithm that uses global self-attention mechanisms without anchors and non-maximum suppression (NMS). RF-DETR Nano provided better mAP for detecting wildlife compared to YOLOv11n, at 0.981 and 0.979, respectively. However, the computational parameter GFLOPS of RF-DETR Nano was 31.5, which is almost five times larger than YOLOv11n. In this study, the trade-off between detection accuracy and computation parameters was evaluated for the object detection algorithms on the validation dataset after training. It was observed that Hjort-YOLO yielded better mAP, precision, recall, and F-1 score compared to the YOLOv11n algorithm. However, the mAP(50–95) was 0.783 by Hjort-YOLO, and YOLOv11n displayed marginally better performance, at 0.796.

Hjort-YOLO demonstrated better mAP than YOLOv11n, the localization and detection accuracy were competitive, while keeping the stricter threshold of mAP(50–95). Hence, the proposed object detection framework Hjort-YOLO showed better and more reliable overall deer detection from the camera traps. The inclusion of the A2C2f-W block in the backbone improved the model's spatial reasoning by using lightweight multi-branch feature extractors to accurately detect wildlife. However, the most significant improvement of the proposed framework was in the computational parameter in GFLOPs, which was the lowest among the benchmarked object detection algorithms. Hjort-YOLO provided 19 percent lower GFLOPS than YOLOv11n, making it even more suitable for real-time animal detection in resource-constrained devices such as Jetson Nano. The confusion matrix from Hjort-YOLO in the validation images showed that the model correctly detected 401 deer instances (TP), 10 detections were identified as FP and 18 instances were missed by the model (FN) compared to the ground-truth bounding boxes during annotation. Hjort-YOLO displayed relatively few incorrect and missed detections compared to the true positive instances. The TP, FP, and FN instances were manually inspected after training the model on the dataset using the validation images.

Table 3 compares computational parameters between the lightweight YOLOv11n detector and the proposed Hjort-YOLO model in terms of accuracy, model complexity, and inference performance. YOLOv11n serves as a strong baseline, achieving an mAP of 0.979 with 2.59 million trainable parameters and a compact 5.5 MB weight file, aligning well with the requirements of low-power or memory-constrained environments. Its inference time is measured at 8 ms, corresponding to a throughput of 21.98 FPS. Hjort-YOLO, despite introducing only a marginal increase in model complexity 2.90 million parameters and a 6 MB weight file provides a measurable performance gain. It achieves a higher mAP of 0.981, indicating improved detection capability. More importantly, Hjort-YOLO provides a substantial reduction in inference time, reaching 6.8 ms per image and a throughput of 24.98 FPS. The addition of the A2C2f-W block in the backbone and the replacement of the C3k2 block with the C2f-W block increased the training parameters. However, the deduction of the SPPF block from the backbone and a detection head reduced the number of layers from the Hjort-YOLO framework, which reduced the number of layers to 143 compared to 181 by YOLOv11n. This improvement signifies an approximate 13.6 percent increase in inference speed over YOLOv11n while maintaining a similarly compact model size. The combined enhancement in accuracy and computational efficiency demonstrates that Hjort-YOLO achieves better architecture-performance optimization, making it particularly suitable for deployment in real-time detection scenarios on edge devices and embedded systems.

**Table 3:** Comparison based on computational parameters on Svanøy deer dataset.

Model	Number of layers	mAP	Training parameters (Million)	Weight file size (MB)	Inference (ms)	FPS
YOLOv11n	181	0.979	2.590035	5.5	8.0	21.98
Hjort-YOLO	143	0.981	2.904306	6.0	6.8	24.98

Svanøy deer dataset consists of both RGB and near infrared images, with 1,432 RGB images and 1,751 near-infrared images. The evaluation of the RGB-only, Infrared-only, and the complete Svanøy deer datasets trained separately using the baseline object detection algorithm Hjort-YOLO is illustrated in Table 4. Both the RGB-only and Infrared-only image datasets converged in earlier epochs than the Svanøy deer dataset, which resulted in an increase in training time. However, it was observed that Hjort-YOLO provided better detection performance compared to RGB-only and Infrared-only images in terms of mAP, mAP(50–95), precision, recall, and F1-score. The combination of both RGB and infrared images provided better exposure of visual diversity in the Svanøy deer dataset to the Hjort-YOLO algorithm for better detection accuracy.

**Table 4:** Performance comparison of Hjort-YOLO trained on RGB, infrared, and Svanøy deer dataset.

Dataset	Epochs	Training time (h)	mAP@50	mAP@50–95	Precision	Recall	F1-score
RGB-only	223	1.689	0.969	0.742	0.942	0.936	0.939
Infrared-only	210	1.822	0.961	0.700	0.956	0.922	0.939
Svanøy deer dataset	289	5.043	0.979	0.796	0.969	0.945	0.957

Table 5 represents an ablation study, which evaluates the contribution of architectural components such as C2f and the A2C2f block in the backbone, the neck, and the detection head reduction from the framework compared to Hjort-YOLO. These blocks were enabled or disabled in Models A and B to assess their impact on the models. The YOLOv11n algorithm has three prediction heads, and it does not have C2f and A2C2f blocks in the backbone and neck of the architecture. YOLOv11n was used as the reference architecture in this ablation study. Model A, maintaining the same backbone architecture as YOLOv11n, achieves an mAP of 0.977, and the reduction of one detection head reduced the GFLOPs significantly to 4.6 from 6.4 compared to YOLOv11n. In Model B, C2f and A2C2f were introduced into the backbone and neck, increasing mAP to 0.979 and GFLOPs to 4.9. The individual contribution of the proposed C2f-W and A2C2f-W blocks was further investigated in Models C and D by maintaining the two-head detection architecture. The third detection head, like YOLOv1n, was retained in Model E by maintaining the C2f-W and A2C2f-W blocks to evaluate the contribution of the prediction head. The Hjort-YOLO architecture incorporating C2f-W, A2C2f-W and detection-head reduction provided an mAP of 0.981, precision of 0.975, Recall of 0.950, and F1-score of 0.962 with the computational complexity of 5.1 GFLOPS. Model E achieved the highest mAP of 0.990, but it required 6.8 GFLOPS. Reducing the detection head responsible for small targets compared to Model E reduced computational parameters by 25 percent in Hjort-YOLO with only a 0.9 percent-point decrement in mAP. These results demonstrated that the proposed C2f-W and A2C2f-W blocks and head reduction provided a favorable trade-off between detection accuracy and computational complexity.

**Table 5:** Ablation study on Svanøy deer dataset.

Model	C2f	A2C2f	Head reduction	mAP	Precision	Recall	F1-score	GFLOPS
YOLOv11n	×	×	×	0.979	0.969	0.945	0.957	6.4
A	×	×	✓	0.977	0.960	0.950	0.955	4.6
B	✓	✓	✓	0.979	0.966	0.945	0.955	4.9
C	C2f-W	×	✓	0.979	0.954	0.945	0.949	4.8
D	×	A2C2f-W	✓	0.965	0.943	0.921	0.932	6.0
E	C2f-W	A2C2f-W	×	0.990	0.979	0.955	0.977	6.8
Hjort-YOLO	C2f-W	A2C2f-W	✓	0.981	0.975	0.950	0.962	5.1

Figure 6 shows the visualization of deer detection by comparing YOLOv11n and Hjort-YOLO on the Svanøy dataset. It can be seen that both of the algorithms provided better results in detecting large target objects in the images than small ones. However, Hjort-YOLO in these examples provided a better confidence score to detect deer compared to the YOLOv11n algorithm. In the RGB images, both of the algorithms detected target objects with proper bounding boxes. Detection was more challenging in the night-vision images, which often contained complex backgrounds due to low illumination and motion blur. These conditions lowered the confidence scores for both algorithms in several cases. The difference between the mAP of YOLOv11n and Hjort-YOLO is minimal, which resulted in the confidence score with minimal improvement while detecting the deer and aligning the bounding boxes. As can be seen in the figure, YOLOv11n displayed a false detection of deer in one of the instances of night vision images, while Hjort-YOLO provided true positive detection.

**Figure 6:**
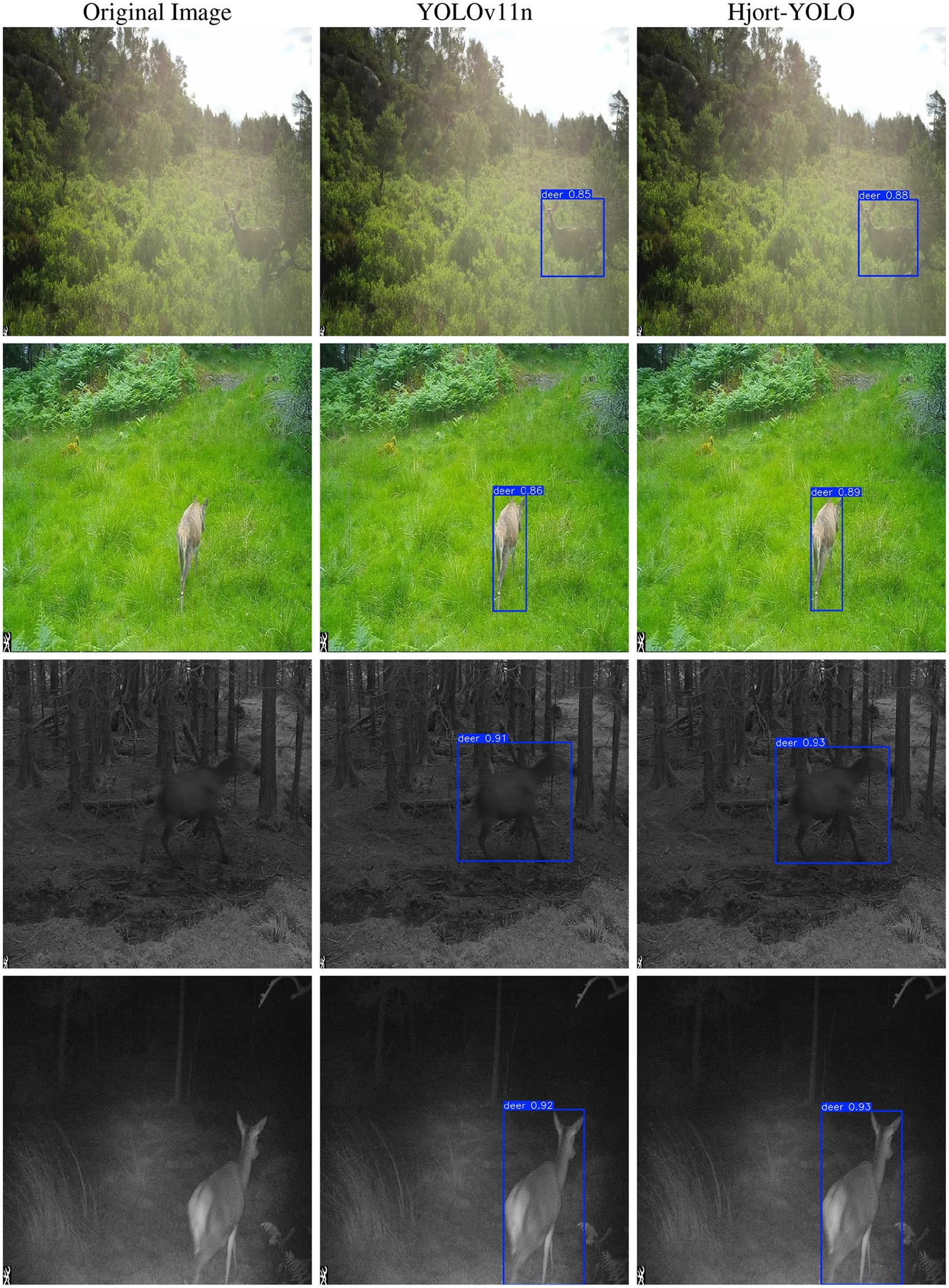
Svanøy deer detection comparison examples using YOLOv11n and Hjort-YOLO (adapted with permission from the ‘deer open dataset Computer Vision Dataset', https://universe.roboflow.com/somethings/deer-open-dataset-8cx4z, licensed under CC-BY 4.0).

### 3.2 Use case B: African wildlife dataset detection

A comparative analysis of the performance of object detection algorithms on the African wildlife dataset is presented in Table 6. A prior work on this dataset by Mamapule et al. (2025) used YOLOv8n. In this study, YOLOv8n was trained in the similar environment to benchmark the object detection parameters with the Hjort-YOLO framework. YOLOv11m provided the best object detection performance compared to the lightweight object detection algorithms. Since YOLOv11m is computationally expensive in terms of training parameters and GFLOPS, the lightweight models will be benchmarked by maintaining the trade-off between detection accuracy and computational complexity. The training was stopped at different epochs due to the threshold value of patience. It was observed that, among the lightweight models, Hjort-YOLO outperformed YOLOv8n and YOLOv11n in terms of mAP, precision, Recall, F1-score and GFLOPS. The African wildlife dataset consists of relatively large animals and less complex background. In the Hjort-YOLO framework, the A2C2f-W and C2f-W blocks extracts the feature maps and pass them through the neck to the detection heads for detecting four different classes of animals. Due to the large pixel size of animals in the images, the low-resolution detection heads of Hjort-YOLO detected and provided better performance compared to the benchmarked lightweight models in Table 6.

**Table 6:** Object detection parameters on the African wildlife dataset.

Model	Epochs	Training time (h)	mAP	mAP (50–95)	Precision	Recall	F1-score	GFLOPS
YOLOv8n (Mamapule et al., 2025)	140	1.162	0.900	0.706	0.895	0.824	0.859	8.2
YOLOv11m	169	2.120	0.937	0.782	0.943	0.868	0.903	68.2
YOLOv11n	148	1.220	0.914	0.718	0.898	0.815	0.854	6.4
Hjort-YOLO	243	1.815	0.922	0.749	0.934	0.849	0.889	5.1

Table 7 compares the object detection algorithms based on object detection parameters across different classes of the African Wildlife dataset. Among YOLOv8n (Mamapule et al., 2025), YOLOv11m, YOLOv11n, and Hjort-YOLO, the best mAP (0.937) was provided by YOLOv11m. However, among the lightweight models, Hjort-YOLO provided better overall mAP than YOLOv8n and YOLOv11n, which were 0.922, 0.90, and 0.914, respectively. YOLOv11n provided better mAP on buffalo (0.939) and rhino (0.95), and Hjort-YOLO provided better mAP on elephant (0.89) and zebra (0.912). In comparison with mAP50–95, Hjort-YOLO provided better performance on rhino (0.804) compared to other lightweight object detection algorithms in Table 7. The zebra had the lowest mAP (50–95) (0.656) compared to other classes performed by different lightweight object detection algorithms. The value of mAP50 is higher than mAP50–95 due to the strict threshold value of the intersection of union (IoU) when comparing predicted bounding boxes with ground truth annotations in mAP (50–95). However, when evaluating class-wise performance, the lowest mAP50–95 value of a class indicates the weakest overall detection quality during the training process. It indicates the challenges in recognizing the zebra class as well as predicting tightly aligned bounding boxes compared to the ground truth. The reasons for the lower mAP (50–95) value for zebra can be the lower number in the training sample, smaller object size, or occlusion in the images. Hjort-YOLO provided better overall and class-wise precision, recall, and F1-score compared to other lightweight object detection algorithms in Table 7.

**Table 7:** Object detection parameter comparison among classes of the African wildlife dataset.

Algorithm	Class	mAP50	mAP50–95	Precision	Recall	F1-score
YOLOv8n (Mamapule et al., 2025)	All	0.900	0.706	0.895	0.824	0.858
	Buffalo	0.898	0.740	0.850	0.843	0.846
	Elephant	0.864	0.669	0.867	0.786	0.824
	Rhino	0.931	0.759	0.922	0.871	0.895
	Zebra	0.905	0.656	0.942	0.798	0.864
YOLOv11m	All	0.937	0.782	0.943	0.868	0.903
	Buffalo	0.936	0.814	0.975	0.862	0.915
	Elephant	0.914	0.755	0.913	0.811	0.858
	Rhino	0.959	0.810	0.941	0.932	0.936
	Zebra	0.938	0.749	0.945	0.868	0.905
YOLOv11n	All	0.914	0.718	0.898	0.815	0.854
	Buffalo	0.939	0.755	0.914	0.838	0.874
	Elephant	0.885	0.677	0.840	0.806	0.822
	Rhino	0.950	0.773	0.910	0.837	0.872
	Zebra	0.881	0.667	0.927	0.780	0.847
Hjort-YOLO	All	0.922	0.749	0.934	0.849	0.889
	Buffalo	0.937	0.768	0.926	0.844	0.883
	Elephant	0.891	0.706	0.887	0.824	0.854
	Rhino	0.948	0.804	0.961	0.872	0.914
	Zebra	0.912	0.716	0.961	0.857	0.906

Figure 7 shows a visual representation of the African Wildlife detection using the YOLOv11n and Hjort-YOLO algorithms. Both algorithms demonstrated effective capabilities in identifying single and multiple animals within the dataset, showcasing robust alignment of bounding boxes and accurate true positive detections. Both YOLOv11n and Hjort-YOLO performed well for detecting single and multiple animals in the dataset. It was observed that the confidence score was increased in Hjort-YOLO compared to the YOLOv11n algorithm. The target animals in the dataset were mostly large. Large animals in the images were detected with a better confidence score by Hjort-YOLO. However, on smaller animals such as the rhino in Figure 8, the confidence score was lower compared to the large rhino in that image, while comparing between YOLOv11n and Hjort-YOLO algorithms. The confidence scores while detecting zebra increased significantly for Hjort-YOLO.

**Figure 7:**
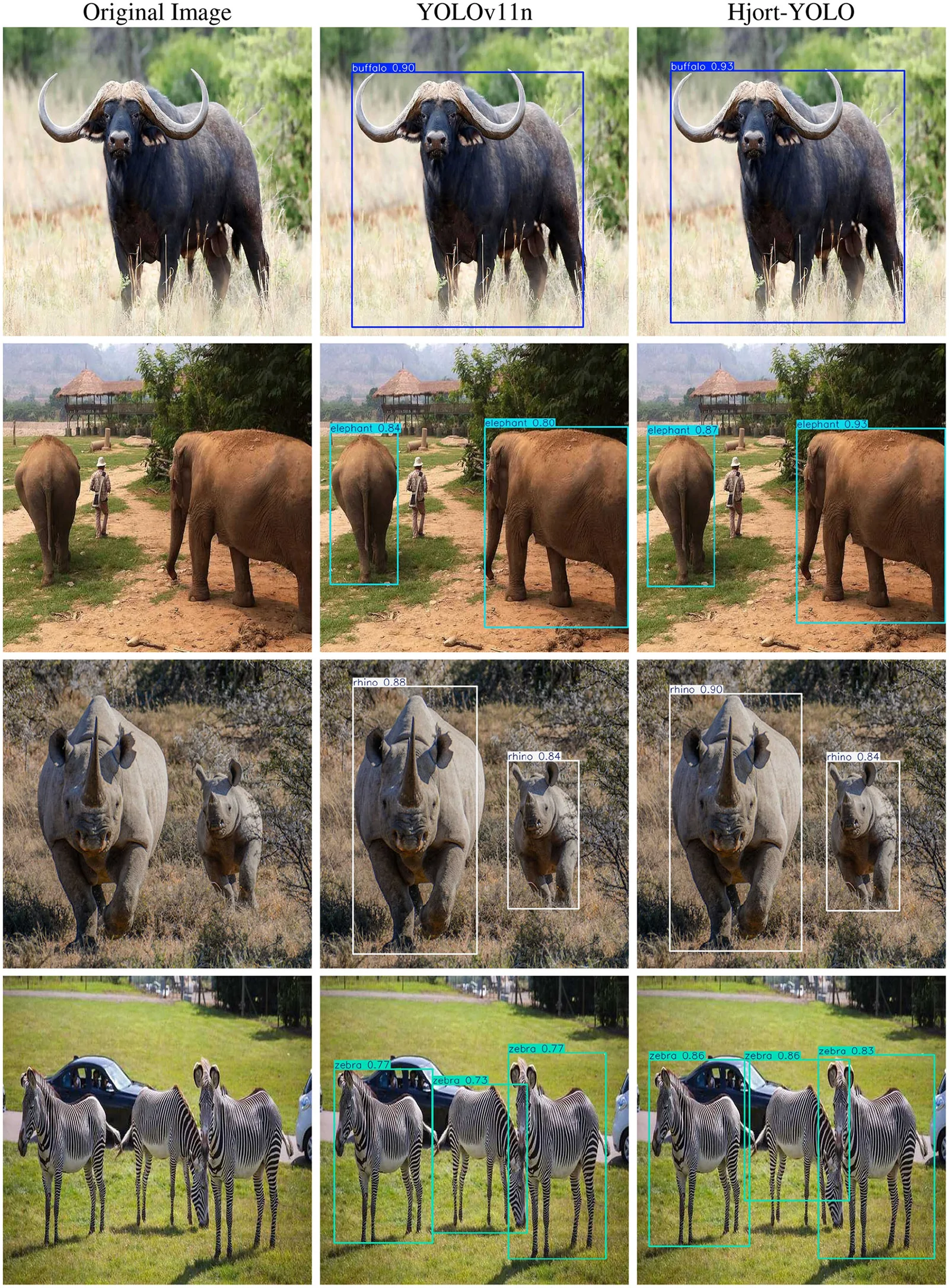
African wildlife detection comparison examples using YOLOv11n and Hjort-YOLO (adapted with permission from Ferreira, B. (2024), https://docs.ultralytics.com/, licensed under the GNU Affero General Public License v3.0).

**Figure 8:**
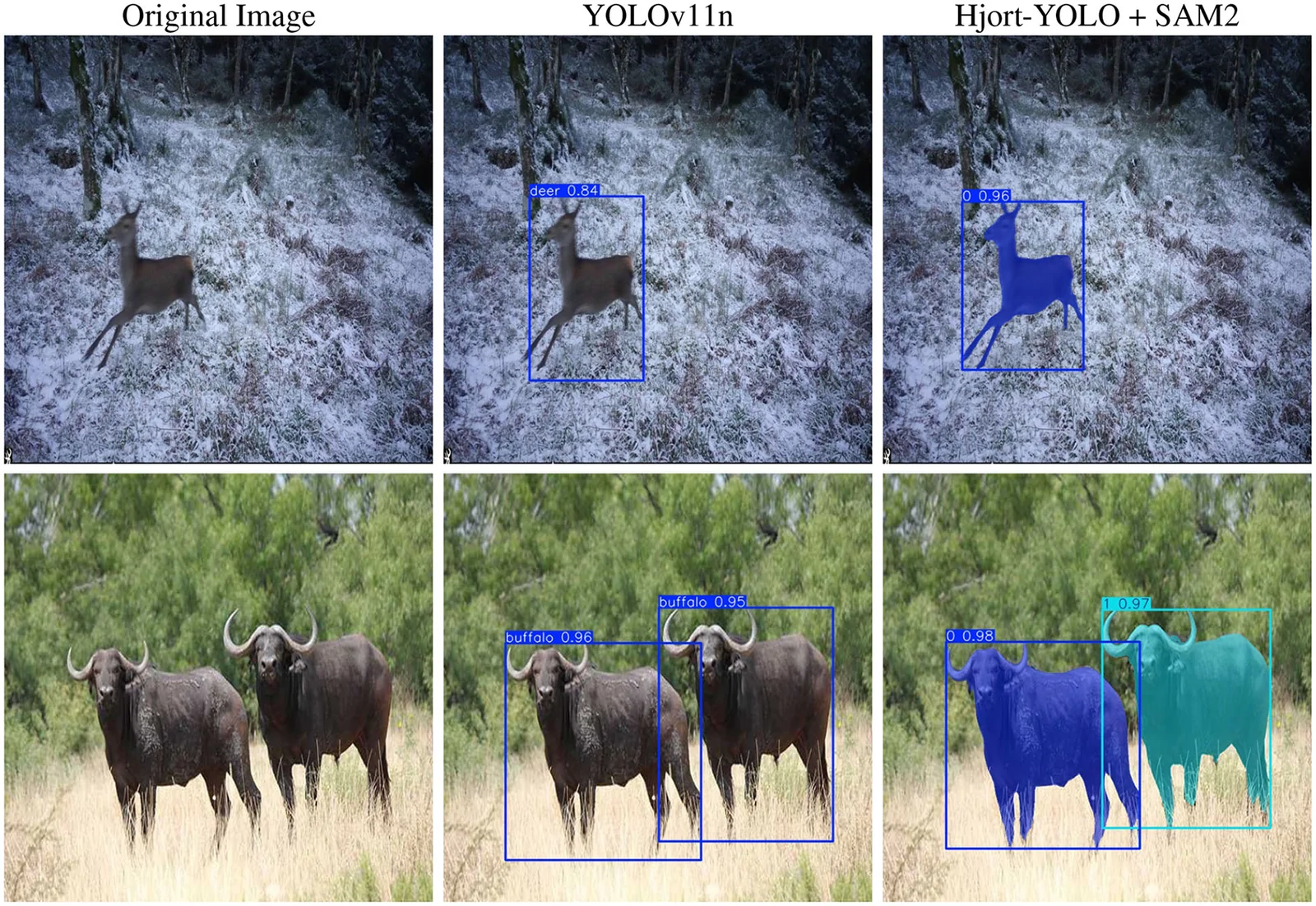
Wildlife segmentation using SAM2 (adapted with permission from the ‘deer open dataset Computer Vision Dataset', https://universe.roboflow.com/somethings/deer-open-dataset-8cx4z, licensed under CC-BY 4.0, and from Ferreira, B. (2024), https://docs.ultralytics.com/, licensed under the GNU Affero General Public License v3.0).

### 3.3 Wildlife visualization with segmentation

The visualization of wildlife detection and segmentation using Hjort-YOLO and SAM2 is shown in Figure 8. The results demonstrate that the wildlife were detected with a high confidence score using Hjort-YOLO, and the bounding boxes were sent through SAM2 to segment the animals. SAM2 successfully segmented the target animals in both wildlife datasets shown in Figure 8. Hjort-YOLO provides bounding boxes and detection confidence score in the image and SAM2 provides more precise spatial information of the target animal about shape and boundaries inside the bounding box after detection. SAM2 also reduces the background noise and ambiguity by isolating the exact region of the target animal. The segmentation helps to distinguish the target wildlife from the background vegetation or any other object by applying a refined object mask. Thus the segmentation using SAM2 along with Hjort-YOLO wildlife detection improves the visualization of the animals.

### 3.4 Wildlife detection visualization with XAI

Figure 9 shows visualizations of the Svanøy deer dataset and the African Wildlife dataset using two algorithms for explainable artificial intelligence (XAI). The performance of Captum was benchmarked with the state-of-the-art XAI tool Grad-CAM. The visualization of the images from both datasets demonstrates the key areas where the detection algorithm focuses for the decision-making process to detect animals. Grad-CAM focuses on the last convolutional layer of Hjort-YOLO before the anchor-free detection head, and a heatmap is generated that highlights the regions for prediction. Hence, Grad-CAM generates a heatmap on the whole image instead of focusing on the bounding box only. Conversely, the Captum tool treats the Hjort-YOLO as a black box, focusing on the framework's output rather than its internal architecture. Captum uses multiple interpretability attribution-based methods, with Integrated-Gradients notable for its visualization (Khan et al., 2024). As Captum looks into the output of the neural network, it gets the bounding box generated by the Hjort-YOLO framework and generates a heatmap on it. The test images were converted to grayscale to improve the heatmap visualization. The A2C2f-W and C2f-W modules in the backbone and neck of the Hjort-YOLO framework generated distinct intermediate feature representations, enabling Captum to propagate feature importance with higher precision within the bounding box. The redundant gradients originating from high-resolution feature maps were reduced in Hjort-YOLO as the third detection head of YOLOv11n (80x80 pixels) was removed. It reduced the significant background noise from the high-resolution feature maps inside the bounding box while visualizing using the Captum tool, reflecting the framework's true decision-making process. Hence, Captum demonstrated better performance in the interpretable visualization of wildlife detection than Grad-CAM when integrating with the Hjort-YOLO framework.

**Figure 9:**
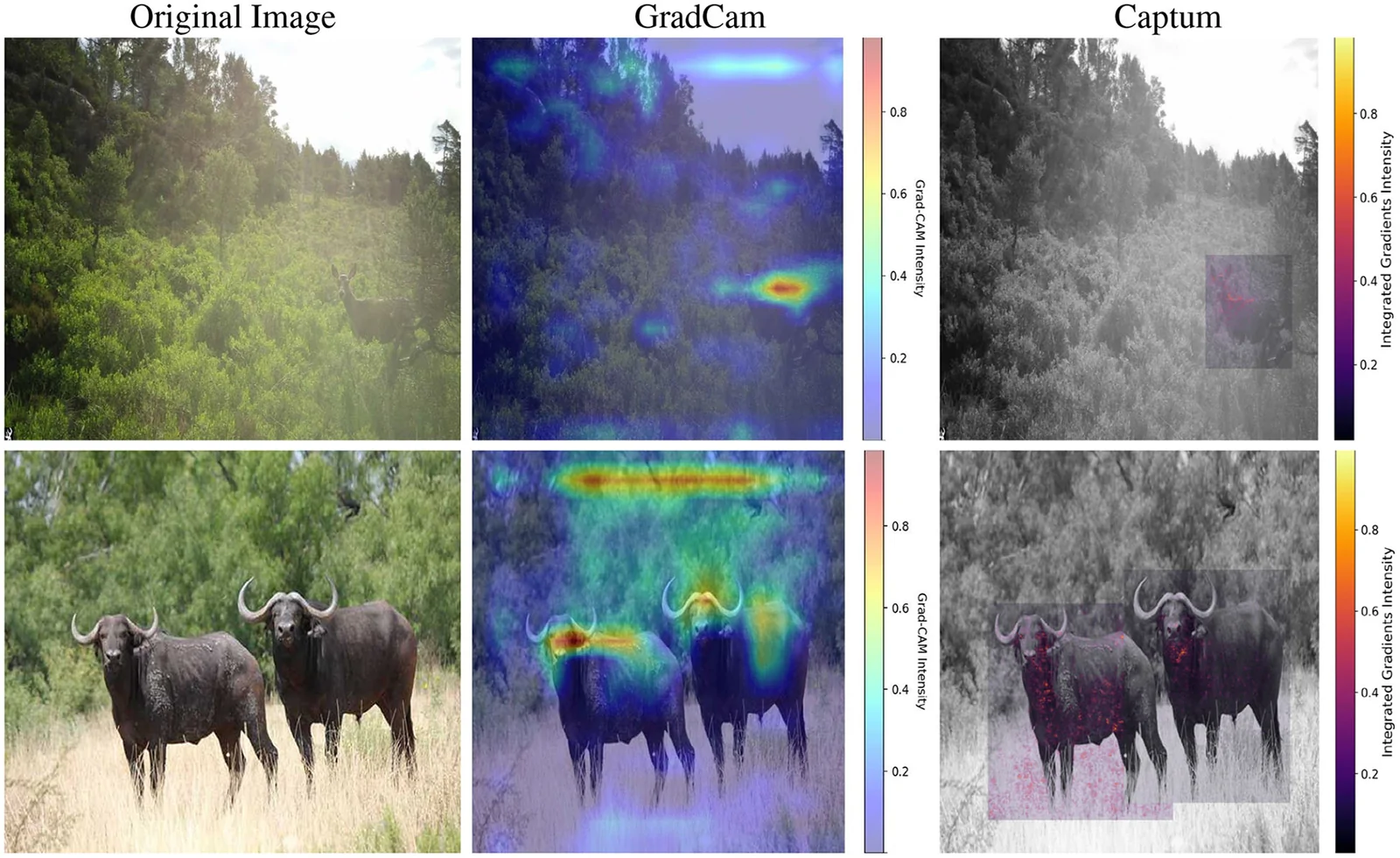
Wildlife visualization using GradCam and Captum with the Hjort-YOLO algorithm (adapted with permission from the ‘deer open dataset Computer Vision Dataset', https://universe.roboflow.com/somethings/deer-open-dataset-8cx4z, licensed under CC-BY 4.0, and from Ferreira, B. (2024), https://docs.ultralytics.com/, licensed under the GNU Affero General Public License v3.0).

## 4 Practical challenges

The Hjort-YOLO framework was designed to detect large target objects in the images since the images were captured using camera traps in the forests. Hence, the algorithm may face challenges in detecting small target objects in the images. The state-of-the-art algorithms provided good detection performance in both the Svanøy deer and the African Wildlife dataset, which makes the algorithm less scalable in terms of improving detection accuracy. The main application for this automated wildlife detection algorithm is to use it in real-world applications for detecting wildlife on the roadside to reduce collisions. The Svanøy deer dataset contains images of deer from an island with forest backgrounds. Since there are no images of wild animals on the roadside, the model may find it challenging to detect roadside animals from embedded devices. However, the model showed good performance in terms of detection accuracy on challenging image backgrounds with blurry and occluded images, which makes the model robust for wildlife detection. The Captum tool provided better interpretability of Hjort-YOLO compared to Grad-CAM tool.

In this study, the end-to-end computational analysis on the Svanøy deer dataset was performed on the Google Colab T4 GPU-based platform. The end-to-end processing time for an image was 607.61 ms using Hjort-YOLO for detection, SAM2 for segmentation, and Captum for explainability. Hjort-YOLO required 0.90 ms for preprocessing, 7.27 ms for inference, and 1.32 ms for post-processing, with a total detection wall time of 10.13 ms. SAM segmentation required 267.10 ms, while the complete XAI stage required 321.69 ms, including 312.71 ms for Captum. The segmentation and XAI stages required a substantial amount of time in the end-to-end computational overhead due to visualization steps. Consequently, Hjort-YOLO showed relatively low computational latency in T4-GPU, which gives an indication of the suitability for deployment on embedded devices for wildlife detection in real-time. The segmentation and explainability processes were computationally intensive and they could be performed on an internet connected edge device or cloud server for detailed visualization and interpretation. However, benchmarking on a physical device such as Jetson Nano is needed for the actual real-time performance, memory requirements, and deployment overhead.

## 5 Conclusion

The proposed Hjort-YOLO algorithm is a robust method for wildlife detection in challenging background conditions from camera-trap images. The algorithm provided a small improvement in terms of detection accuracy compared to state-of-the-art algorithms for detecting the wildlife images from the Svanøy deer and the Wildlife African animal dataset, while demonstrating a significant improvement in detection time and FPS. The modification in the backbone and the detection head provided the trade-off between detection precision and computational complexity. The algorithm was based on YOLOv11n, given its suitability for implementation in embedded devices aimed at real-time detection applications. In this study, the Hjort-YOLO algorithm demonstrated better computational parameters than the current state-of-the-art lightweight algorithms by increasing 0.2 percent and 13 percent in terms of mAP and FPS, and reducing 17 percent of inference time. The low computational complexity makes it suitable for implementing in embedded devices such as Jetson Nano or Raspberry Pi to detect wild animals on the roadside to reduce animal-vehicle collisions.

In the future, the model will be implemented on an embedded device, with the goal to detect wildlife in Norway, especially deer, as part of exploratory work on an alert system for drivers to reduce animal-vehicle collisions. We aim to lower the model's computational complexity to make it better suited for embedded devices. Besides, in future works, the architecture of the algorithm will be explored and improved using a transformer-based model, such as RF-DETR, for real-time detection with minimal trade-off between detection accuracy and computational parameters. We also aim to explore object tracking across multiple frames and combine the model output with other animal detection modalities to improve the system's reliability further.

## Data Availability

The original pictures and data of Svan8y deer dataset can be obtained from the authors upon request. The African Wildlife animal dataset can be downloaded from https://github.com/ultralytics/assets/releases/download/v0.0.0/african-wildlife.zip. The code and detailed instructions for the workflow will be available on GitHub (https://github.com/mominhvl/HjortYOLO). The raw data supporting the conclusions of this article will be made available by the authors, without undue reservation.
